# The relationship between body image and sexual satisfaction in women who have undergone hysterectomy

**DOI:** 10.1590/1806-9282.20240776

**Published:** 2024-09-30

**Authors:** Elmas Gökçe, Sevda Karakaş

**Affiliations:** 1 Istanbul Arel University, Graduate Education Institute – İstanbul, Turkey.; 2 Gümüşhane University, Faculty of Health Sciences, Department of Nursing – Gümüşhane, Turkey.

**Keywords:** Hysterectomy, Body image, Sexual satisfaction, Women

## Abstract

**OBJECTIVE:**

This study aims to examine the relationship between body image and the level of sexual satisfaction in women who have undergone a hysterectomy.

**METHODS:**

This descriptive study utilized a relational screening model. Conducted between June 2023 and March 2024, the study included a total of 300 sexually active women who had undergone a hysterectomy 6 months prior. Data were collected using a personal information form, the Golombok-Rust Sexual Satisfaction Scale, and the Body Image Scale through face-to-face interviews lasting approximately 20–30 min.

**RESULTS:**

The average age of the participating women was 44.4±10.55 years. Of the participants, 96.7% were married and 42.4% had entered menopause. The average score on the Body Image Scale was 81.2±3.8, and the average score on the Golombok-Rust Sexual Satisfaction Scale was 33.0±15.1. A positive significant relationship was found between body image and sexual satisfaction (p<0.05). There was also a statistically significant positive relationship between the total scores on the Body Image Scale and the Golombok-Rust Sexual Satisfaction Scale with age and frequency of sexual intercourse (p=0.049, p<0.001, p<0.001, p=0.047, and p=0.014). It was determined that as the age and frequency of sexual intercourse increased, the levels of body image and sexual satisfaction also increased.

**CONCLUSION:**

Our study found that women’s self-image is negatively affected, and their level of sexual satisfaction decreases following a hysterectomy.

## INTRODUCTION

The uterus is often considered a symbol of femininity, sexuality, fertility, and motherhood for many women. It is associated with concepts such as childbirth, menstruation, youth, and attractiveness, making the loss of the uterus synonymous with the loss of womanhood^1,2,3^. Hysterectomy, the surgical removal of the uterus or its lining, is a significant medical intervention for women^1,2^. This surgery impacts the four crucial elements of sexual health: body image, gender role function, sexual functions, and reproductive ability, all of which can be adversely affected^3^. Hysterectomy is one of the most commonly performed surgical procedures worldwide, including in Turkey, where it is estimated that over 200,000 women undergo the procedure annually^4^. In the United States, the prevalence of hysterectomy among women aged 35–55 years is approximately 16.3%, while in Canada, this rate exceeds 30%. Studies have reported higher hysterectomy rates among women with lower education and income levels^3,5,6^. In Australia, one in three women undergo a hysterectomy by the age of 60 years, with 30% of these women also having both ovaries removed during the procedure^6^. Hysterectomy can lead to physical and biological effects such as disruption of body integrity and loss of fertility. It can also result in negative body image, psychosexual issues, and social issues due to the perceived loss of femininity^7^.

In addition to the removal of the uterus, the blood vessels and, particularly, the nerves that supply the uterus and other genital organs can be damaged, which adversely affects sexual life. It has been reported that women who have undergone a hysterectomy experience increased sexual dysfunction due to loss of nerve tissue, reduced blood flow, decreased lubrication related to the loss of the cervix, and the negative effects of scar tissue^8^. Post-hysterectomy, a woman’s sexuality is a critical issue that requires attention^9^. For the quality of sexual life and sexual satisfaction to be at the desired level, a normal endocrine system, intact innervation, and adequate blood flow to these organs are necessary^6,4,10^. For these reasons, the changes that occur in the pelvic structure after a hysterectomy negatively impact women’s sexual lives.

Following a hysterectomy, women are at risk for numerous physical and psychological issues, in addition to the impact on their sexual lives. Notably, younger women who undergo a hysterectomy are more likely to experience negative body image, anxiety, and depression compared to older women^11^. This study aims to examine the relationship between body image and the level of sexual satisfaction in women who have undergone a hysterectomy.

## METHODS

This study is a descriptive research employing a relational screening model. Power analysis was used to determine the sample size, with a power of 99%, an alpha value of 0.05, and an effect size of 0.25. The target was set at 259 women who had undergone a hysterectomy. Our study was conducted between June 2023 and February 2024, and 300 women who had undergone a hysterectomy agreed to participate. Women who had undergone a hysterectomy 6 months prior were included in the study to assess the effects of the surgery on body image and sexual life. Data were collected using a personal information form, the Golombok-Rust Sexual Satisfaction Scale (GRISS), and the Body Image Scale (BIS). The data collection process was completed through face-to-face interviews.

### Research questions

Hysterectomy negatively impacts body image and the level of sexual satisfaction. In line with this primary purpose, the following questions were addressed:

- What is the level of sexual satisfaction in women who have undergone a hysterectomy?
- What is the body image of women who have undergone a hysterectomy?
- Is there a relationship between body image and the level of sexual satisfaction in our study?

### Data collection tools

Three data collection tools were used in the study: a personal information form, the GRISS, and the BIS.

The BIS consists of 40 items, each related to an organ or part of the body (e.g., arm, leg, and face) or a function (e.g., sexual activity level). This scale was developed in Turkish by Hovardaoğlu in 1993, with item-test correlations ranging from r=0.45 to r=0.89. In this study, Cronbach’s alpha coefficient for the BIS was determined to be r=0.91^12^.

The GRISS is a 28-item, 5-point Likert-type scale designed to measure the quality and functionality of an individual’s sexual relationship. The GRISS was adapted into Turkish by Tuğrul et al. in 1993, with a Cronbach alpha coefficient of 0.91. In this study, Cronbach’s alpha values for the subdimensions of the GRISS ranged from 0.584 to 0.748^13^.

### Statistical analysis

The data were analyzed using the SPSS 21 software. Descriptive statistics, including frequency, percentage, mean, standard deviation, maximum, and minimum values, were used in the analysis. The normality of the data distribution was assessed using the Shapiro-Wilk test. Since the data followed a normal distribution, independent sample t-tests and one-way ANOVA tests were employed for analysis. Bonferroni and Games-Howell tests were used as post hoc tests. Statistical significance was set at p<0.05.

### Ethical aspects of research

Ethical approval was obtained from the Ethics Committee of Istanbul Arel University. Institutional permission was granted by Istanbul Prof. Cemil Taşçıoğlu City Hospital (Approval date: 26.05.2023, number: 11). The principles of the Declaration of Helsinki were adhered to throughout the study.

## RESULTS

The average age of the women who participated in the study was 44.4±10.55 years. Of the participants, 96.7% were married and 42.4% had entered menopause (Table 1). Before undergoing a hysterectomy, 52.5% of the women sought medical consultation due to excessive bleeding, 19.4% due to menstrual irregularities, and 5.4% due to pain. It was found that 52.3% of the women had their first sexual intercourse approximately 3 months after the surgery, 43.3% around 2 months after, and 3.7% about 1 month after the surgery. Post-surgery, 58.7% of the women reported engaging in sexual intercourse 1–2 times per week, 23.3% 1–2 times per month, and 18% 3–4 times per week. Additionally, 16.3% of the women reported that their marriages were negatively affected post-surgery. The average score on the BIS was 81.2±3.8, and the average score on the Sexual Satisfaction Scale was 33.0±15.1.

**Table 1:** Distribution of socio-demographic characteristics of the women (n=300).

Characteristics	Number (n)	Percentage (%)
Age (years)		
<25	7	2.3
26–35	12	4.0
36–45	150	50.0
>45	131	43.7
Employment status		
Yes	67	22,3
No	233	77.7
Marital status		
Married	290	96.7
Single	10	3.3
Duration of marriage (years)		
1–5	32	10.6
6–10	40	13.3
11–20	75	25,0
20 years and over	153	51.1

The study found a significant positive relationship between body image and overall sexual satisfaction, frequency, communication, satisfaction, avoidance, touch, and anorgasmia. As the level of body image increased, so did the levels of overall sexual satisfaction, frequency, communication, satisfaction, avoidance, touch, and anorgasmia (p<0.05).

Following a hysterectomy, the frequency of sexual intercourse was found to have a statistically significant relationship with the BIS (p=0.011), the Frequency subscale of the Sexual Satisfaction Scale (p<0.001), Communication (p<0.001), Satisfaction (p<0.001), Avoidance (p<0.001), Touch (p<0.001), Vaginismus (p=0.008), Anorgasmia (p<0.001), and the Total score (p<0.001). The Anorgasmia subscale scores of the Sexual Satisfaction Scale were significantly higher in participants who engaged in sexual intercourse 1–2 times per week post-surgery compared to those who had intercourse 3–4 times per week or less than 1–2 times per month (p<0.001, p=0.008) (Table 2).

**Table 2:** Investigation of the relationship between Body Image Scale and Sexual Satisfaction Scale by frequency of sexual intercourse (n=300).

Frequency of sexual ıntercourse	Mean	SD	Min	Max	Median	p
Body Image Scale (BIS)						
Weekly 3–4 times	81.6	8.1	73	116	80	0.011
Weekly 1–2 times	83.5	4.7	80	94	80	
Monthly 1–2 times or less	80.6	2.6	76	105	80	
Frequency						
Weekly 3–4 times	3.8	1.9	0	8	4	<0.001
Weekly 1–2 times	4.1	1.2	0	7	4	
Monthly 1–2 times or less	3.6	2.1	0	8	4	
Communication						
Weekly 3–4 times	2.8	1.9	0	8	2	<0.001
Weekly 1–2 times	4.3	1.8	0	8	4	
Monthly 1–2 times or less	3.6	1.9	0	8	4	
Satisfaction						
Weekly 3–4 times	3.4	1.5	0	7	4	<0.001
Weekly 1–2 times	5.7	1.3	2	9	6	
Monthly 1–2 times or less	4.3	2.4	0	14	4	
Avoidance						
Weekly 3–4 times	2.1	3.4	0	12	0	<0.001
Weekly 1–2 times	7.7	2.5	0	14	8	
Monthly 1–2 times or less	3.2	3.5	0	14	2	
Touch						
Weekly 3–4 times	4.9	3.7	0	15	3	<0.001
Weekly 1–2 times	9.0	2.7	3	16	8	
Monthly 1–2 times or less	5.6	4.4	0	16	4	
Vaginismus						
Weekly 3–4 times	6.4	3.3	2	13	4	0.008
Weekly 1–2 times	7.0	3.0	3	16	7	
Monthly 1–2 times or less	5.4	2.7	2	16	4	
Anorgasmia						
Weekly 3–4 times	4.2	3.5	0	11	3	<0.001
Weekly 1–2 times	8.8	2.1	3	14	8	
Monthly 1–2 times or less	4.8	3.1	0	15	4	
Total score						
Weekly 3–4 times	27.9	13.8	5	71	24.5	<0.001
Weekly 1–2 times	46.6	8.8	22	65	46	
Monthly 1–2 times or less	30.5	14.7	9	75	28.5	

There was a statistically significant positive relationship between age and the scores on the BIS and the subscales of the Sexual Satisfaction Scale, including Frequency, Satisfaction, Anorgasmia, and the Total score (p=0.049, p<0.001, p<0.001, p=0.047, and p=0.014). As age increased, so did the levels of body image, sexual satisfaction, frequency, satisfaction, and anorgasmia (Table 3).

**Table 3:** nvestigation of the relationship between the Body Image Scale and Sexual Satisfaction Scale by age (n=300).

	Age	
	r	p
Body Image Scale (BIS)	0.114	0.049
Sexual Satisfaction Scale (GRISS)		
Frequency	0.348	<0.001
Communication	0.045	0.440
Satisfaction	0.202	<0.001
Avoidance	0.071	0.217
Touch	0.095	0.100
Vaginismus	-0.020	0.735
Anorgasmia	0.115	0.047
Total score	0.142	0.014

## DISCUSSION

The study found that in women who underwent a hysterectomy, as the level of body image increased, so did the overall sexual satisfaction, frequency, communication, satisfaction, avoidance, touch, and anorgasmia levels. Similar to our findings, a study conducted on 188 women who had undergone a hysterectomy at the oncology center outpatient clinic of Mansoura University in Egypt also found that as body image improved, so did the level of sexual satisfaction^14^. Another study examining the relationship between marital adjustment, body image, and sexual satisfaction in women who had undergone a hysterectomy found that as body image improved, so did the levels of sexual satisfaction^15^. Hassan et al. also investigated the relationship between sexual function, body image, and depression in 60 women who had undergone a hysterectomy in Egypt, and their findings indicated a significant positive relationship between body image and sexual functions^16^.

Contrary to the results of our study, Till et al. conducted a cohort study on women who underwent hysterectomy for benign indications and found that the frequency of sexual intercourse and the quality of sexual life did not change post-hysterectomy^17^. Similarly, in the study by Körpe et al., it was reported that women’s sexual lives were not adversely affected after both abdominal and laparoscopic hysterectomy surgeries^18^. The size of the incision site on women’s body after surgery and possible increases in healing time may decrease the perception of body image and may lead to sexual reluctance, lack of self-confidence, and sexual dissatisfaction. Buhur et al. reported that laparoscopic hysterectomy is a safer and convenient minimally invasive surgical procedure with advantages such as faster recovery, earlier discharge, and earlier return to professional and social life^19^. Similarly, Tormena et al. reported the advantages of single-port laparoscopic hysterectomy compared to multi-port laparoscopy, including shorter recovery time, lower incidence of infection, less postoperative pain, and higher postoperative patient satisfaction scores. They stated that these advantages after single-port laparoscopy may provide better postoperative outcomes compared to multi-port laparoscopy by reducing surgical tissue trauma^20^. In this study, it was observed that the overall sexual satisfaction, frequency, satisfaction, and vaginismus levels were higher in employed women who had undergone a hysterectomy compared to unemployed women who had undergone the same surgery. Contrary to our findings, other studies have found that unemployed women who had undergone a hysterectomy reported higher levels of sexual satisfaction than their employed counterparts. For instance, research conducted by Mohammadi-Zarghan and Ahmadi reported that unemployed women who had undergone a hysterectomy had higher levels of sexual satisfaction and its subdimensions compared to employed women^15^.

The study also revealed that the level of body image was statistically significantly higher in women who had sexual intercourse 3–4 times per week post-surgery compared to those who had intercourse 1–2 times per month or less. Previous studies in the literature have indicated that as the frequency of sexual intercourse increases, so does the level of sexual satisfaction^21,22^. Our study determined that younger women experienced a more significant decrease in body image and sexual satisfaction levels compared to older women. It was found that younger women had a considerable reduction in body image and sexual satisfaction levels. This result might be because hysterectomy is more commonly performed in older women, and the impact of age and menopause on sexual functions could be a natural consequence^23^.

## CONCLUSION

Our study found that women’s self-image is negatively affected, and their level of sexual satisfaction decreases following a hysterectomy. It is essential to assess body image and sexual life, which are significant determinants of quality of life, and to identify the needs of women post-hysterectomy. Randomized controlled trials with high evidence value are recommended.

### Limitations and strengths of the study

Sexuality is an important problem in Turkish society, especially in developing countries. The limited number of studies examining sexual problems after hysterectomy in the literature reveals the originality and strengths of this study. The limitations of the study include the fact that the study was conducted in a single center and the sample size was small.

## Data Availability

The data that support the findings of this study are available from the corresponding author upon reasonable request.
